# Identifying Perimenopausal Symptoms in Women Diagnosed With Depression: A Focused Audit at a Tertiary Care Hospital

**DOI:** 10.1192/bjo.2024.572

**Published:** 2024-08-01

**Authors:** Naima Gul

**Affiliations:** Institute of Psychiatry Rawalpindi Medical University, Rawalpindi, Pakistan

## Abstract

**Aims:**

This audit aimed to assess the recognition and management of perimenopausal symptoms in women diagnosed with depression at the Psychiatry Outpatient Department (OPD) of Benazir Bhutto Hospital, Pakistan. It focused on identifying gaps in screening for perimenopausal symptoms among these patients.

**Methods:**

Conducted over a year, this retrospective audit included 250 women aged 45–55 years, previously diagnosed with depression. Post-diagnosis screening for perimenopausal symptoms was performed using the Menopause-Specific Quality of Life Questionnaire (MENQOL) and the Greene Climacteric Scale. Data on initial diagnostic criteria, treatment modalities, and patient outcomes were reviewed. Follow-up interviews provided insights into ongoing symptom management and treatment satisfaction.

**Results:**

The retrospective screening revealed that 78% of these women had significant perimenopausal symptoms per the Greene Climacteric Scale, which were initially overlooked. MENQOL results showed 65% experiencing a substantial impact on quality of life due to menopausal symptoms. Treatment primarily consisted of antidepressants (used by 82% of patients), while 8% received psychological counseling, and 10% were advised on lifestyle adjustments and non-hormonal therapies. Only 45% of the patients reported satisfactory symptom management, indicating a potential discrepancy between the treatments for depression and the underlying perimenopausal condition.

**Conclusion:**

The audit at Benazir Bhutto Hospital demonstrates a high incidence of undiagnosed perimenopausal symptoms in women treated for depression, suggesting a critical need for improved screening protocols. The results indicate that integrating perimenopausal symptom assessment into the initial diagnostic process for depression could lead to more effective, individualized treatment strategies. This approach may enhance the overall treatment satisfaction and quality of life for perimenopausal women, underscoring the importance of holistic patient care in psychiatric settings.

